# Metformin loaded non-ionic surfactant vesicles: optimization of formulation, effect of process variables and characterization

**DOI:** 10.1186/2008-2231-21-7

**Published:** 2013-01-11

**Authors:** Anchal Sankhyan, Pravin K Pawar

**Affiliations:** 1 Chitkara College of Pharmacy, Chitkara University, Chandigarh-Patiala Highway, Rajpura, Patiala, Punjab, 140401, India

**Keywords:** Anti-diabetic, Niosomes, Metformin, Dicetyl phosphate, Sustained release

## Abstract

**Background:**

Metformin an oral hypoglycemic has been widely used as a fist line of treatment of Type II Diabetes but in a very high dose 2–3 times a day and moreover suffers from a number of side effects like lactic acidosis, gastric discomfort, chest pain, allergic reactions being some of them. The present work was conducted with the aim of sustaining the release of metformin so as to decrease its side effects and also reduce its dosing frequency using a novel delivery system niosomes (non-ionic surfactant vesicles). Non-ionic surfactant vesicles of different surfactants were prepared using thin film hydration technique and were investigated for morphology, entrapment, in-vitro release, TEM (transmission electron microscopy) and physical stability. Optimized formulation was further studied for the effect of Surfactant concentration, DCP (Dicetyl phosphate), Surfactant: cholesterol ratio and volume of hydration. The release studies data was subjected to release kinetics models.

**Results:**

The prepared vesicles were uniform and spherical in size. Optimized formulation MN3 entrapped the drug with 84.50±0.184 efficiency in the vesicles of the size 487.60±2.646 and showed the most sustained release of 73.89±0.126. Also it was resulted that 100 molar concentration of cholesterol and surfactant, Presence of DCP, equimolar ratio of span 60: cholesterol and 15 ml of volume of hydration were found to be optimum for miosome preparation.

**Conclusions:**

The present work concluded metformin loaded niosomes to be effective in sustaining the drug release leading to decreased side effects and increased patient compliance.

## Background

With the advent of therapeutics, oral delivery is the most widely used and the most convenient route of drug delivery. Despite of phenomenal advances in other dosage routes viz. injectable, inhalable, transdermal, nasal, oral delivery still remain well ahead of the pack as the preferred delivery route. Higher concentration is focused in making the oral formulations viable if it is not immediately viable, than in plumping for an alternative delivery method. The top 50 drugs selling in the world have 84% oral delivery [1]. Variety of approaches have been tried to enhance the oral bioavailability of poorly soluble drugs using the excipients with approved or GRAS (generally regarded as safe) status. Micronization by spray-drying, freeze-drying, crystallization and milling; nanosizing into nanoparticles by various techniques with high-pressure homogenization being one of most efficient; crystal engineering of polymorphs, hydrates, solvates, co-crystals, supercritical fluid and sonocrystallization; solid dispersions developed by melt-mixing, solvent evaporation, supercritical fluid and melt extrusion; solubilizing ability of cyclodextrins; solid lipid nanoparticles prepared by high-pressure homogenization and microemulsion technology and other colloidal drug delivery systems including emulsions, microemulsions, self-emulsified and self-microemulsified drug delivery systems, liposomes etc. have been widely researched for enhancement of oral bioavailability [2]. Other attempts of enhancing oral bioavailability include Solid lipid nanoparticles, mucoadhesive delivery system, lipid digestion models, Supersaturatable Formulations, nanoemulsion, nanocapsules, fast-dispersing dosage forms and pH-sensitive supramolecular assemblies [3-10].

Diabetes is a group of chronic carbohydrate metabolism disorders resulting from diminished or absent action of insulin by altered secretion, decreased insulin efficacy or combination of both the factors leading to hyperglycemia. Type II diabetes is the most common type counting about 90-95% of the diagnosed cases, characterized with normal or even excess of insulin levels with insulin resistance being the major cause of increased glucose levels [11]. Metformin a biguanide enhances insulin sensitivity, found to be effective in impaired glucose tolerance, obese patients and patients with cardiovascular diseases and is used as first line of drug in treatment of Type II diabetes [12].

The oral bioavailability of Metformin is 50-60% as it is BCS (Biopharmaceutical Classification System) class III drug and has site-specific absorption in the GI tract [13]. The drug has negligible plasma protein binding, relatively short half life of 1.5-4.5 hours and requires administration of 500 mg dose two or three times a day [14]. Moreover the drug suffers from serious but rare side effects of lactic acidosis with 50% mortality, chest pain, allergic reactions accompanied by high incidences of concomitant gastrointestinal symptoms such as diarrhea, abdominal discomfort, vomiting, stomachache, headache and lethargy [15]. Researchers endeavored for years to enhance the oral bioavailability, sustain the drug release for better patient compliance and reduced side effects of the most widely used oral hypoglycemic Metformin. The conventional dosage form of Metformin i.e. tablet has been modified by various approaches to get the desired results. Matrix tablets with sustained release have been prepared using hydroxypropyl methyl cellulose as a hydrophilic polymer, hydrophilic synthetic polymers and hydrophobic natural polymers and by incorporation of lipophillic waxes by melt granulation [14,16]. With the view to enhance patient compliance taste masked tablets and oro-dispersible tablets have also been formulated, also the FDA (Food and Drug Administration) has approved metformin-glipizide tablets for oral suspension, metformin-glyburide oral solution and linagliptin-metformin hydrochloride tablets [17-19]. Niosomes are non ionic surfactant vesicles having lamellar structure formed by self assembly of surfactant molecules. To improve the oral bioavailability of poorly water soluble drug like griseofulvin, the noisome (vesicular) system was developed [20]. In another report, the polysaccharide coated noisomes of propranolol HCl was developed for the oral drug delivery and studied the effect of polysaccharide cap using hydrophobic anchors on the non ionic surfactant vesicles [21]. The only novel delivery system so far utilized for the delivery of metformin is mucoadhesive ispaghula-sodium alginate beads [22]. Niosomes have been used to deliver a number of drugs and have shown pronounced benefits of enhanced bioavailability, sustained release, targeted delivery, decreased side effects, high stability, easy modification, and so on [23]. In the present investigation niosomes have been prepared to enhance oral bioavailability of class III antidiabetic drug. The nonionic surfactant vesicles have been prepared and evaluated for entrapment efficiency, in vitro drug release, particle size, zeta potential, TEM. Also the effect of various parameters viz. molar concentration and molar ratio of cholesterol and surfactant, presence of DCP and volume of hydration was studied on the various evaluated parameters. The studied system is developed for efficient treatment of Type II diabetes.

## Methods

Metformin was a kind gift sample from Matrix Laboratories (Hyderabad, India). Cholesterol was supplied by Fisher Scientific (Mumbai, India). The non-ionic surfactants viz. Span 20, Span 40, Span 60, Span 80, Tween 20, Tween 80 and Brij 30 were purchased from Loba Chemie Pvt. Ltd. (Mumbai, India). Tween 60 was procured from Sisco Research Laboratories Pvt. Ltd. (Mumbai, India) and HPLC chloroform was provided by Merck Specialities Pvt. Ltd. (Mumbai, India). All the ingredients used in the procedures were of analytical grade.

### Preparation of niosomes

The nano sized vesicles were prepared using Thin Film Hydration Method. The specified quantities of cholesterol, non-ionic surfactant and Dicetyl Phosphate (DCP) were completely dissolved in 10 ml HPLC chloroform contained in a clean and dry Round Bottom Flask (Table 1). The transparent solution was reduced to a thin dry film using Rotary Vaccum Evaporator (Perfit, India) at 50.00±2.00°C. Metformin was dissolved in phosphate buffer pH 6.8 and the thin dry film was hydrated using this buffered drug solution. The film is allowed to hydrate for about 1 hour for the formation of niosomes [22]. Milky dispersion is prepared which is kept at 4°C for 24 hours for maturation of the formed vesicles.

**Table 1:** Composition (molar ratio), entrapment efficiency and particle size of metformin niosomes

**Formulation code**	**Cholesterol**	**Surfactant**	**Dicetyl phosphate (mg)**	Entrapment* efficiency (%)	Particle size* (nm)
MN1	250	Span 60 (250)	5	85.16±0.12	574.33±2.08
MN2	250	Span 60 (250)	-	87.12±0.05	636.00±2.65
MN3	100	Span 60 (100)	5	84.50±0.18	487.60±2.65
MN4	100	Span 60 (100)	-	86.51±0.15	504.66±2.52
MN5	75	Span 60 (75)	5	83.66±0.08	388.0±3.61
MN6	75	Span 60 (75)	-	84.71±0.05	496.66±3.51
MN7	250	Span 40 (250)	5	83.91±0.01	565.33±2.52
MN8	250	Span 40 (250)	-	85.07±0.08	624.67±2.08
MN9	100	Span 40 (100)	5	83.71±0.12	462.33±2.08
MN10	100	Span 40 (100)	-	84.88±0.11	496.33±1.53
MN11	75	Span 40 (75)	5	84.56±0.08	378.67±1.53
MN12	75	Span 40 (75)	-	86.63±0.02	484.67±1.53

*Mean ± SD, *n* = 3.

### Entrapment efficiency

The entrapment efficiency of the matured niosomes was determined using centrifugation method. The measured volume 5 ml of the prepared dispersion was centrifuged using cooling centrifuge (RIS-24BL, REMI India) at 6°C for 1 hour to separate the free drug from niosomes. The niosomes formed a cake floating at the top of tube and clear solvent containing the unentrapped drug remained at the bottom. The cake was resuspended in 5 ml phosphate buffer pH 6.8 and the process was repeated twice by centrifugation for 30 minutes to ensure complete removal of free drug. After suitable dilution with Phosphate buffer pH6.8 the clear fraction was used for the determination of free drug spectrophotometrically by UV-visible Spectrophotometer (AU-2701, Systronics, Mumbai, India) [24]. The entrapment efficiency was calculated using the formula

$$\text{PercentEntrapmentEfficiency}=\frac{\text{InitialDrug}\left(D_{i}\right)-\text{Unentrappeddrug}\left(D_{u}\right)}{\text{InitialDrug}\left(D_{i}\right)}X100$$

### In-vitro drug release

The in-vitro drug release pattern was studied using modified USP dissolution apparatus Ι. Samples were placed on the dialysis membrane previously soaked overnight in phosphate buffer pH 6.8 and attached to lower end of a glass tube. The tubes were immersed in dissolution vessel containing phosphate buffer pH 6.8 maintained at 37±0.5°C. Samples were withdrawn at regular interval of time and replaced with equal amount of buffer to maintain sink condition [25]. The samples were analyzed by UV/Visible spectrophotometer (AU-2701, Systronics, Mumbai, India) for the drug release pattern. The study was continued upto 8 hours.

### Particle size and zeta potential

The particle size of the non-ionic surfactant vesicles was determined by dynamic light scattering technique also known as photon correlation spectroscopy using Zetasizer Nano ZS-90 (Malvern Instruments Ltd., UK) [26]. The samples diluted suitably by filtered water (0.5 micrometer filter- Himedia) were placed in the cuvettes and the procedure was carried out at 90° angle and temperature 25°C to determine the size of the particles in the range of 0.6nm to 3 microns. The zeta potential was determined using combination of laser Doppler velocimetry and phase analysis light scattering by Zetasizer Nano ZS-90 (Malvern Instruments Ltd.; UK). The diluted niosome dispersions were located in zeta meter cell for determination of electrophoretic mobility [27]. All the experiments were performed in triplicate.

### Transmission electron microscopy

The morphology of the vesicles was examined by tramsmission electron microscopy (TEM). A drop of niosomal dispersion was diluted 10 times and was stratified onto a carbon-coated copper grid for 1 minute and excess was removed by filter paper. A drop of 2% phosphotungstic acid solution was stratified to stain the vesicles; excess was removed by a tip of filter paper and left to air dry. The grid was observed by transmission electron microscopy (Hitachi, H-7500) and by using imaging viewer software the images were analyzed and captured [28].

### Drug release kinetic data analysis

The release data obtained from various formulations was studied further for fitness of data in different kinetic models like Zero order, First order, Higuchi and Korsmeyer-Peppas release models [29].

### Physical stability testing

Physical stability of the niosomes was studied by leaching of the drug from the vesicles in the native prepared form i.e. dispersion stored under refrigeration. The optimized dispersion with the composition of cholesterol and span in 100:100 molar ratio with DCP was sealed in glass vials and stored under refrigeration temperature (2-8°C) for a period of 90 days. Samples were withdrawn at definite intervals of time and the amount of drug remaining was calculated by the method employed for entrapment efficiency determination [30].

## Results

Span 20, Span 80, Tween 20, Tween 60, Tween 80 and Brij 30 did not formed thin and dry film at the round bottom flask so were not used further in the study. Thereby Span 40 and Span 60 were used for the preparation and evaluation in the study.

### Morphology

The prepared niosomal solution was a homogeneous dispersion and after maturation of 24 hours was studied under the microscope for morphological evaluation. The vesicles were uniform, spherical in shape with traces of aggregation. The formulations prepared with inclusion of DCP were found to be free from aggregation.

### Entrapment efficiency

The amount of drug loaded in the vesicles was determined by centrifugation method which separates the entrapped and the unentrapped drug. The percent entrapment efficiency was calculated and was found to be in the range of 83.66±0.08-87.12±0.05 (Table 1).

### In-vitro release

**Figure 1:**
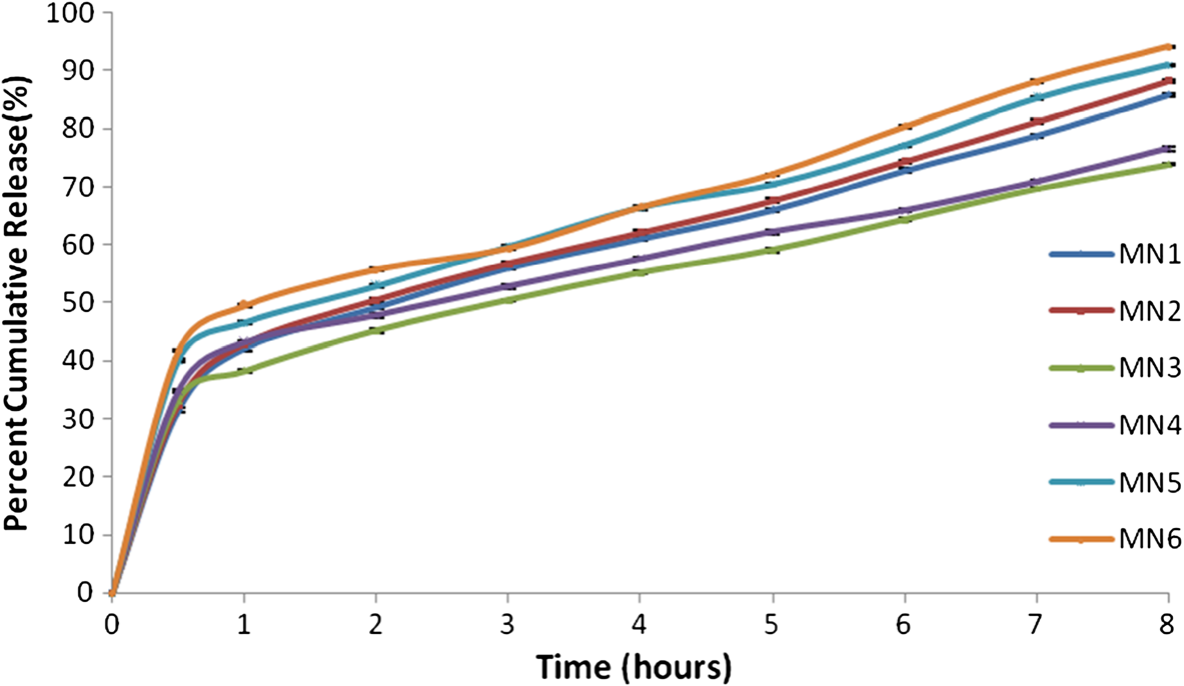
In vitro drug release pattern of metformin niosomes using Span 60.

**Figure 2:**
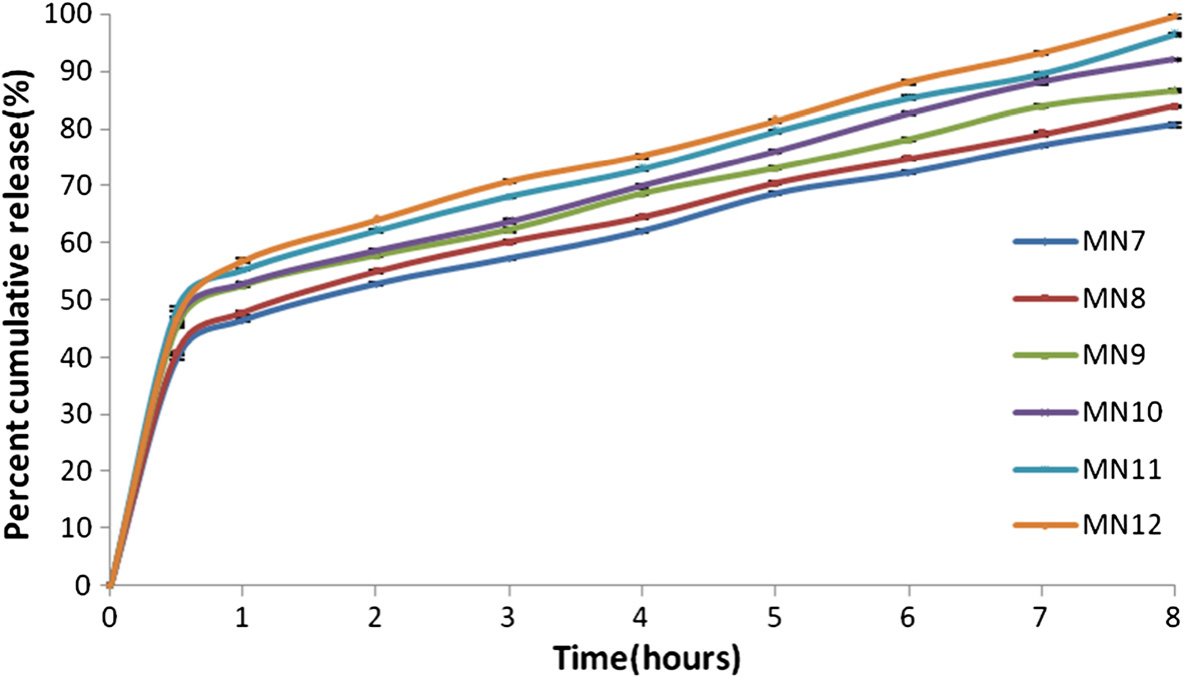
In vitro drug release pattern of metformin niosomes using Span 40.

The release pattern of the drug from the niosomes was studied using modified USP dissolution apparatus Ι. The formulations presented sustained release upto 8 hours with MN3 having the most sustained release of 73.89±0.13 at the end of 8 hours. On the basis of most sustained release and sufficient entrapment of 84.50±0.18 MN3 was selected as the optimized formulation (Figure 1) (Figure 2).

### Particle size and zeta potential

The average vesicular size of niosomes of all the batches was measured in the range of 388.0±3.61-644.67±2.08 (Table 1). Also the surface charge was studied by zeta potential measurement. The niosomes were found to be stabilized by large negative values of zeta potential and the polydispersity index (PDI) was 0.123.

### Transmission electron microscopy

**Figure 3:**
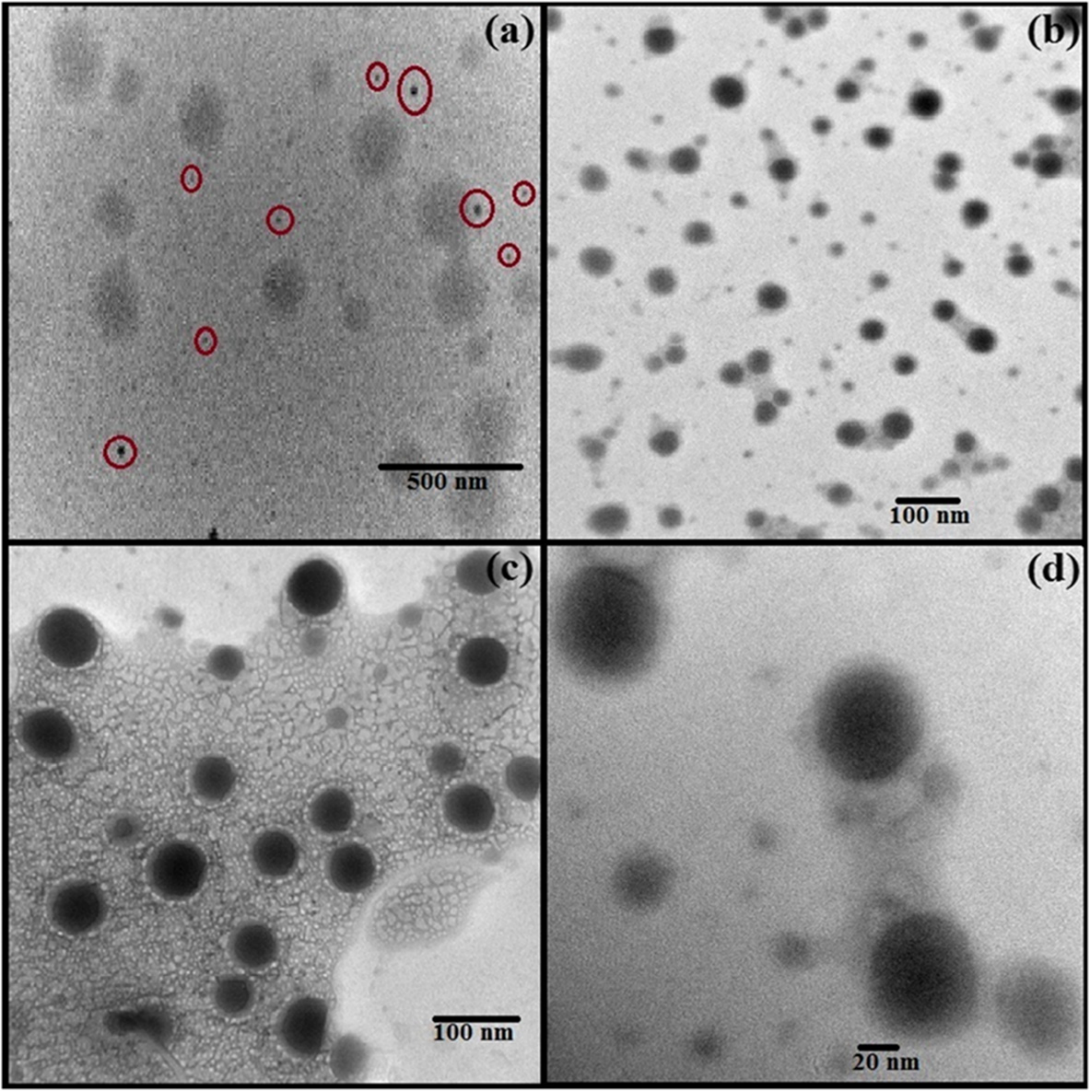
TEM images (a) Uniform distribution; (b) Uniform spherical shape & size; (c) Visible membrane & dark core; (d) Uniform shape.

The TEM photomicrographs clearly indicate that vesicles were uniform, unilamellar and spherical in shape. Also the positive staining showed the drug to be concentrated in the core of the vesicles with drug free bilayer (Figure 3).

### Release kinetics

The study of drug release kinetics showed that in all the formulations the best fit model was found to be Korsmeyer-Peppas with ‘n’ smaller than 0.45 suggesting the Fickian diffusion release mechanism for the drug. Formulation MN3 showed the lowest release of 73.89±0.13 in 8 hours and had correlation coefficient (r =0.996) (Table 2).

**Table 2:** Drug release kinetics profile of metformin niosomes

**S. NO**	**Formulation code**	**Zero order**		**First order**		**Higuchi**		**Korsemeyer - Peppas**		
		r^2^	K	r^2^	K	r^2^	K	r^2^	K	n
1	MN1	0.851	24.00	0.566	0.137	0.973	7.98	0.993	1.620	0.267
2	MN2	0.855	24.35	0.564	0.139	0.973	7.97	0.995	1.629	0.264
3	MN3	0.809	23.66	0.541	0.135	0.953	9.75	0.996	1.580	0.261
4	MN4	0.779	25.86	0.506	0.142	0.937	11.51	0.968	1.629	0.204
5	MN5	0.824	27.81	0.511	0.150	0.954	11.07	0.975	1.660	0.252
6	MN6	0.821	28.82	0.500	0.153	0.946	11.76	0.968	1.601	0.336
7	MN7	0.760	28.96	0.474	0.151	0.927	13.51	0.992	1.664	0.203
8	MN8	0.759	29.94	0.469	0.153	0.928	13.90	0.998	1.676	0.216
9	MN9	0.734	32.70	0.440	0.160	0.909	16.13	0.960	1.714	0.184
10	MN10	0.767	32.54	0.454	0.161	0.925	15.19	0.969	1.717	0.195
11	MN11	0.754	34.53	0.440	0.165	0.920	16.47	0.991	1.739	0.198
12	MN12	0.769	34.65	0.450	0.165	0.932	15.79	0.992	1.751	0.202
13	MN13	0.812	30.27	0.487	0.156	0.943	12.70	0.957	1.692	0.214
14	MN14	0.814	23.35	0.538	0.135	0.939	9.86	0.959	1.589	0.203
15	MN15	0.793	25.36	0.509	0.141	0.929	11.30	0.952	1.624	0.190
16	MN16	0.702	37.27	0.405	0.170	0.883	19.50	0.963	1.766	0.156
17	MN17	0.820	23.06	0.546	0.134	0.948	9.46	0.988	1.579	0.223
18	MN18	0.820	24.69	0.535	0.139	0.954	9.91	0.976	1.606	0.235

*r^2^=regression coefficient, K= release rate constant, n=diffusional exponent.

### Physical stability

The formulation investigated for physical stability studies showed the residual drug content in the niosomes to be 77.61± 0.22 at the end of three months. The results concluded that almost 99% of the drug was retained upto 1 month and at the end of the study 91.84% drug was retained by the formulation (Table 3).

**Table 3:** **Stability of optimized metformin niosome formulation under refrigeration temperature (2-8**°**C) storage condition**

**Time (days)**	Entrapment efficiency* (%)	Drug remaining* (%)
0	84.50±0.19	100.00±0.00
7	84.36±0.10	99.83±0.12
15	84.24±0.13	99.68±0.15
30	83.91±0.08	99.30±0.09
45	83.39±0.10	98.69±0.12
60	81.55±0.21	96.50±0.25
90	77.61±0.22	91.84±0.26

*Mean ± SD, *n* = 3.

## Discussion

### Effect of type of surfactants

The effect of non-ionic surfactant was studied on the entrapment efficiency, particle size and in-vitro release of the prepared niosomes. Span 60 and Span 40 were the two surfactants used in the study and the formulations were prepared by varying their concentrations to the same extent. Span 60 preceded Span 40 in terms of in-vitro release and entrapment efficiency by presenting the most sustained release in all the prepared batches and higher entrapment efficiencies when compared to corresponding formulations (Figure 1) (Figure 2). Span 60 and Span 40 has the same head group and differs in the chain length of the alkyl chain which determines their performance. Span 60 having longer chain length provide more stable vesicles contributing to the higher entrapment and delayed release. Also the ordered gel state and higher phase transition temperature offered by Span 60 plays a significant role in the observed outcomes [31,32]. The size of the vesicles was slightly high in formulations prepared by Span 60 at the same molar concentration of Span 40. This may be the repercussion of the longer alkyl chain and its stronger interactions with cholesterol molecules which resulted larger core space providing larger entrapment [33]. Decreased molar concentrations of surfactant and cholesterol reduced the entrapment of the drug and the size which may be the aftermath of the insufficient bilayer material to form a strong membrane and to encapsulate the drug efficiently. The case was not similar for the drug release an optimized amount was required for the controlled effect and increase or decrease in the amount of surfactant lead to early drug loss (Table 1).

### Effect of DCP

Charge inducer used was also scrutinized to corroborate its effects on the characteristics of the vesicles. The negative charge induced by DCP resulted in smaller size of the vesicles leading to lower entrapment efficiency (Table 1). Charge on the surface of the bilayer cause repulsive forces which compel the vesicles to be more curved and smaller in sizes. Smaller the size smaller will be the volume enclosed in the core and smaller will be the amount of drug entrapped. DCP was also found to have an advantageous impact in retarding the drug release rate which may be attributed to the stability provided by it to the membrane [34] (Figure 1) (Figure 2). DCP has reported effects of providing integrity and uniformity and preventing aggregation and fusion which have been established in the study by the maintenance of sustained release and evident reduction of aggregation in photomicrographs and visual observations [35]. Aggregation has also been lowered by sonication of the prepared dispersion clearly visible in photomicrographs of the vesicles.

### Effect of surfactant: cholesterol ratio

The surfactant: cholesterol ratio used, plays a decisive determinant of the properties and the behavior of the bilayer of the niosomes. So to predict the consequences, different molar ratios of surfactants were incorporated into different batches and were investigated for the parameters of particle size, entrapment and in-vitro release. The evaluation proposed equimolar ratio to be the most apt for development of a superior delivery system providing all the required features. Surfactant is the core material for bilayer formation, but the bilayer of just the non-ionic surfactant is not strong enough to serve as host for the drug. So cholesterol having a steroidal rigid structure provides the required strength to the bilayer, despite of the fact that cholesterol itself is incapable of layer formation [36]. When the molar ratio of the surfactant was increased from 75:100 to 125:100 in context with cholesterol the entrapment enhanced to an optimum level and then declined, here the optimized ratio was equimolar i.e. 100:100. The excess of cholesterol at lower ratio tend to disrupt the regular bilayer structure leading to lower entrapment and higher drug release caused by leakage. Irregularities in the membrane structure added by the excess of cholesterol may have lead to increase in the vesicular size. With the increased ratio of the surfactant the entrapment has also improved to an optimized level as the excess of cholesterol is compensated by the added surfactant. At the molar ratio of 100:100 the surfactant and the cholesterol have the best fit arrangement in the bilayer serving to be efficient drug carrier. As the cholesterol acts as the rigidizing agent having the ability to cement the leakages, its deficiency upon increasing the ratio of the surfactant after the optimized ratio lead to the formation of leakage points resulting in lower entrapment and increased drug release [37]. The particle size has decreased with the increasing concentration of surfactant, may be due to bilayer formation with decreased cholesterol content. MN14 being the optimized formulation has highest entrapment of 84.14±0.07, with the most sustained release of 74.37±0.22 after 8 hours and the smallest particle size of all 481.33±3.06 nm (Table 4).

**Table 4:** Effect of surfactant: cholesterol ratio on entrapment efficiency, particle size and in vitro drug release

**Formulation code**	**Cholesterol:surfactant (Span 60)**	**Volume of hydration (ml)**	Entrapment efficiency* (%)	Particle size* (nm)	In vitro drug release* (%)
MN13	100:75	15	78.84±0.09	524.33±3.06	94.18±0.50
MN14	100:100	15	84.14±0.07	481.33±3.06	74.37±0.22
MN15	100:125	15	79.27±0.04	445.33±2.08	79.03±0.10
MN16	100:100	10	66.92±0.33	490.67±2.52	94.03±0.14
MN17	100:100	15	83.37±0.09	477.33±2.08	74.60±0.33
MN18	100:100	20	79.36±0.10	455.0±2.64	78.31±0.20

*Mean ± SD, *n* = 3.

### Effect of volume of hydration

Hydration of the thin dry film by the aqueous media is a critical step and the volume used determines the resulting features of the niosomes. The drug used being hydrophilic in nature was dissolved in the buffer and this solution was used for hydration. So the volume also altered the concentration obtained. The results showed no specific pattern of relationship between volume and the evaluated parameters. MN17 prepared using 15 ml of hydrating media served to be best fit formulation with highest entrapment and most retarded release. Any increase or decrease in volume hampered the entrapment and subsequently the release (Table 4). The possible reason for such a behavior could be stated as 15 ml providing ample space for vesicle formation. The lower volume may have lead to incomplete or distorted formation of bilayer due to excess of material and less of space. Whereas in the case of larger volume, the material have been dispersed in large volume with large interacting area. This may have caused formation of smaller vesicles with decreased entrapment efficiency.

The in vitro release revealed prolonged delivery of the drug for about 8 hours which suffice the daily requirement in Type II diabetes treatment. The sustained release is also proven to decrease the side effects resulting from higher drug concentrations or presence of drug at the areas other than the target site [38]. Also the patient compliance is enhanced by use of niosomes with the possibility of once daily dose as compared to 2–3 doses per day. The negative zeta potential exhibit the negative charge developed on the surface causing the repulsive forces preserving the formulation from aggregation and fusion of vesicles thereby maintaining their integrity and uniformity [35]. The stability testing imply minimal drug lose from the vesicles upto 3 months at refrigeration temperature. In the light of above facts niosomes have been found to meet the requirements for successful delivery of the drug. Furthermore successful encapsulation of the drug in niosomes has also opened the doors for exploitation of other benefits presented by this novel delivery system viz. enhanced penetration, targeted delivery, reduced dose, protection from harsh environment etc.

## Conclusions

The investigation conclusively supported niosomes to be an advantageous drug delivery system with high degree of entrapment and sustained release of the drug over extended period of time. The results also concluded that the studied variables have a significant impact on the entrapment and release of drug from the niosomes. Also the Molar concentration and molar ratio of cholesterol and surfactant, the charge inducer DCP and the volume of hydration used should be in optimized value and greatly influence the entrapment of drug in the vesicles and also alters the performance of niosomes. The optimized formulation (cholesterol: surfactant, 100:100 molar concentration) with DCP (5 mg) and 15 ml volume of hydration showed the most sustained release of drug and was found to be the best formulation. The careful control of all the above factors allow the production of a dosage form with sustained release capable of combating the side effects and also reducing the dosing frequency with the greater patient compliance. Suggesting metformin loaded niosomes to be an efficient drug carrier system in treatment of Type II Diabetes.

## Abbreviations

DCP: Dicetyl Phosphate; TEM: Transmission Electron Microscopy.

## Competing interests

The author(s) declare that they have no competing interests.

## Authors’ contributions

All authors have read and approved the final manuscript.
